# Prediction of Mesiodistal Width of Unerupted Lateral Incisors, Canines and Premolars in Orthodontic Patients in Early Mixed Dentition Period

**Published:** 2016-11

**Authors:** Mohammad Hossein Toodehzaeim, Alireza Haerian, Ali Alesaeidi

**Affiliations:** 1 Associate Professor, Department of Orthodontics, Faculty of Dentistry, Shahid Sadoughi University of Medical Sciences, Yazd, Iran; 2 Assistant Professor, Department of Orthodontics, Faculty of Dentistry, Shahid Sadoughi University of Medical Sciences, Yazd, Iran; 3 Dentistry Student, Faculty of Dentistry, Shahid Sadoughi University of Medical Sciences, Yazd, Iran

**Keywords:** Dentition, Mixed, Dentition, Permanent, Tooth, Unerupted

## Abstract

**Objectives:**

Proper diagnosis and prevention of malocclusion are superior to treatment. Discrepancy between arch length and tooth size in mixed dentition period is a condition requiring timely diagnosis. Estimating the mesiodistal width of unerupted teeth according to the size of erupted ones can lead to earlier diagnosis of malocclusion. On the other hand, the best timing for serial extractions is before the eruption of lateral incisors. The aim of this study was to present prediction formulas for mesiodistal width of unerupted lateral incisors, canines and premolars in an Iranian population based on the width of erupted permanent mandibular central incisors and maxillary first molars.

**Materials and Methods:**

A total of 120 dental models (60 males, 60 females) of orthodontic patients between 11–25 years were evaluated in Yazd city. The measurements were made by a digital caliper on the widest mesiodistal width of teeth at the interproximal contacts. Data were analyzed to calculate the prediction equation.

**Results:**

The prediction equation in the upper jaw was y=0.57x+10.82 for males, y=0.7x+6.37 for females and y=0.64x+8.46 for both sexes. The equation for the lower jaw was y=0.76x+2.86 for males, y=0.74x+3.53 for females and y=0.77x+2.7 for both sexes.

**Conclusions:**

The prediction equations suggested in this study can predict the mesiodistal width of unerupted lateral incisors, canines and premolars in an Iranian population in early mixed dentition period without taking radiographs.

## INTRODUCTION

Proper diagnosis and prevention of malocclusion are superior to treatment; thus, detection of a potential malocclusion is the best orthodontic service, which can be provided in early mixed dentition period [1,2]. Accurate examination is mandatory for comprehensive treatment planing; therefore, a series of diagnostic records are collected as a supplement to clinical examination as baseline records [3]. Diagnostic analysis of dental models is an important step in making a diagnosis along with taking medical and dental history, clinical examination and radiography [3]. Many malocclusions appear in the mixed dentition period, in the age range of six to 12 years, and a proper intervention at this time may be able to prevent or decrease the intensity of these malocclusions [4]. Tooth size/arch size discrepancy is a condition that requires early diagnosis and treatment. It accounts for a large percentage of malocclusions and is observed in the mixed dentition period. Mixed dentition analysis is critical for early diagnosis of malocclusion [4,5]. The size of canines and premolars is estimated in mixed dentition analysis [6]. The Moyers and Tanaka-Johnson are the most popular analyses, which require the mesiodistal width of the four lower incisors. These methods are the results of studies on Caucasian ethnic groups; therefore, they might not be very accurate for other ethnicities [5]. Estimating the mesiodistal width of the unerupted canines and premolars according to the size of erupted teeth (central incisors and first molars) can lead to earlier diagnosis of malocclusion [5].

On the other hand, a good timing for serial extractions is before the eruption of lateral incisors. The aim of this study was to present prediction equations for estimation of the mesiodistal width of unerupted lateral incisors, canines and premolars in an Iranian population based on the width of erupted teeth.

## MATERIALS AND METHODS

A total of 120 dental models (60 males, 60 females) of orthodontic patients between 11–25 years, who were visited in private offices in Yazd city were evaluated. Sample size was calculated considering α=0.05, β=0.2, r=0.6 and 80% study power. The inclusion criteria were: (1) age under 25 years to decrease physiological attrition effect, which occurs with age (attrition affects not only the occlusal surface but also proximal surfaces and decreases the mesiodistal width of teeth); (2) All permanent teeth that needed to be measured (incisors, canines, premolars and first molars) had to be present in the models; (3) the diagnostic record evaluation should not show any reduction in mesiodistal width of teeth (due to attrition, caries, fracture or restorations), congenital defects or impression errors and (4) dental models should not have malformed or defective teeth. The largest mesiodistal width of the mandibular central incisors and maxillary first molars, lateral incisors, canines and premolars of both jaws was measured using a digital caliper (Mitutoyo, Tokyo, Japan) with 0.001mm accuracy for each model. Individual measurement error was evaluated by re-measuring 20 random cases by the same clinician one week later and another clinician afterwards, and the mean values were compared and analyzed by intraclass correlation coefficient. Finally, the linear regression was used to acquire equations for prediction of the sum of the widths of lateral incisors, canines and first and second premolars in each jaw. The regression equation was presented as Y = a + bX. The constants “a” and “b” were calculated for both sexes combined and for males and females separately. In the above-mentioned equation, Y stands for the sum of the widths of lateral incisors, canines and first and second premolars in one quadrant and X stands for the sum of lower central incisors and upper first molars. The data were analyzed using SPSS version 20 (SPSS Inc., IL, USA).

## RESULTS

The mean age was 15.71±2.34 years in males and 15.5±2.09 years in females. Table 1 shows the mean mesiodistal width of mandibular central incisors and maxillary first molars in both males and females and also shows the mean mesiodistal width of lateral incisors, canines and premolars of both jaws in both sexes. The Pearson’s correlation coefficient showed that there was a significant correlation between the mesiodistal width of the mandibular central incisors and maxillary first molars with the mesiodistal width of the lateral incisor, canine and premolars of one quadrant (P≥0.001). This correlation was stronger in the mandible and in males (Table 2). The prediction equation for the upper jaw was y=0.57x+10.82 for males, y=0.7x+6.37 for females and y=0.64x+8.46 for both sexes (Table 3). The equation for the lower jaw was y=0.76x+2.86 in males, y=0.74x+3.53 in females and y=0.77x+2.7 in both sexes (Table 4). According to the intraclass correlation coefficients, the reliability scales for all variables were more than 0.90 with a P-value of less than 0.001, which showed excellent reliability of the results.

**Table 1:** Descriptive statistics of the sum of mesiodistal widths of teeth (mm)

** Teeth **	** Sex **	** Mean **	** Standard deviation **	** Median **	** Maximum **	** Minimum **
** Permanent mandibular central incisors and maxillary first molars **	Male	32.29	1.56	32.26	35.94	27.54
	Female	31.62	1.00	31.56	34.28	29.10
	Both	31.96	1.35	31.95	35.94	27.54
** Lateral incisors, canines and premolars in one side of the upper jaw **	Male	29.20	1.31	28.92	32.51	26.56
	Female	28.46	1.27	28.49	31.15	26.06
	Both	28.83	1.34	28.65	32.51	26.06
** Lateral incisors, canines and premolars in one side of the lower jaw **	Male	27.62	1.56	27.97	30.82	24.26
	Female	27.00	1.16	26.98	29.86	24.72
	Both	27.31	1.41	27.26	30.82	24.26

**Table 2:** The Pearson correlation coefficient between mesiodistal widths of teeth (mm)

** Teeth width **	** Sex **	** Sum of lateral incisor, canine and premolar widths in one side of upper jaw **	** Sum of lateral incisor, canine and premolar widths in one side of lower jaw **
** Permanent mandibular central incisors and maxillary first molars **	Male	R ^*^ =0.68; P<0.001	R=0.7; P<0.001
	Female	R=0.55; P<0.001	R=0.64; P<0.001
	Both	R=0.64; P<0.001	R=0.74; P<0.001

* R=Pearson correlation coefficient

**Table 3:** Regression parameters for prediction of the sum of widths of lateral incisors, canines and premolars in one side of the upper jaw from the sum of widths of permanent mandibular central incisors and maxillary first molars

** Teeth width **	** Sex **	R square ^*^	** P-value **	** Standard error of the estimate **	** Regression factors for prediction of lateral incisor, canine and premolar widths in one side of upper jaw **	

					** A **	** B **
** Permanent mandibular central incisors and maxillary first molars **	Males	0.46	0.000	0.973	10.82	0.57
	Females	0.30	0.000	1.073	6.37	0.70
	Both	0.41	0.000	1.032	8.46	0.64

* R square: determination coefficient

**Table 4:** Regression parameters for prediction of the sum of widths of lateral incisors, canines and premolars in one side of lower jaw from the sum of widths of permanent mandibular central incisors and maxillary first molars

** Teeth width **	** Sex **	R square ^*^	** P-value **	** Standard error of the estimate **	** Regression factors for prediction of lateral incisor, canine and premolar widths in one side of lower jaw **	

					** A **	** B **
** Permanent mandibular central incisors and maxillary first molars **	Males	0.58	0.000	1.01	2.86	0.76
	Females	0.41	0.000	0.90	3.53	0.74
	Both	0.54	0.000	0.95	2.70	0.77

* R square: determination coefficient

## DISCUSSION

Most mixed dentition analyses estimate the mesiodistal width of canines and premolars. One of these analyses is the Moyer's technique, which uses mesiodistal width of lower incisors [3,7]. These methods have been established by studies on the Caucasian ethnic group; therefore, they might not be accurate for other ethnicities [3,5]. The samples in this study were between 11–25 years of age. Mittar et al, [8] and Tikku et al, [9] also used the same age range. This is because the teeth required for this analysis erupt after 11 years of age and physiological attrition has not yet affected the proximal surfaces of the teeth at this age range. Malocclusions, which require extraction, should be diagnosed as soon as possible; therefore, estimating the mesiodistal width of lateral incisors, canines and premolars after the eruption of lower central incisors and maxillary first molars can be very helpful for treatments such as serial extraction.

According to the results of this study, the mean mesiodistal width of lateral incisors, canines and premolars is larger in males compared to females. Flores-Mir et al, [10] Diagne et al, [11] Yuen et al, [12] and Peng et al, [13] also found similar differences between males and females. The reason for this difference is the larger average body size of males compared to females, which affects tooth size. Ling and Wong [4] and Verzi et al, [14] also presented equations for each sex.

The permanent lower central incisors and the maxillary first molars were used in this study. Boboc and Dibbets [15] and Mittar et al, [8] used the same approach. Ling and Wong [4], Tanaka and Johnston [6], Al-Khadra [16], Lee-Chan et al, [17] Moyers [7], Diagne et al, [11] and Peng et al, [13] also used central incisors of the mandible to predict the mesiodistal width of unerupted canines and premolars. Cattaneo et al, [18] and Toodehzaeim et al, [19] predicted the mesiodistal width of canines and premolars using maxillary first molars. This was due to their eruption before all other teeth. The mandibular central incisors and maxillary first molars were used for prediction in this study, which was similar to studies by Boboc and Dibbets [15], Cattaneo et al, [18] and Toodehzaeim et al, [19]; the reason for this choice was to have data from both jaws. The prediction formulas were more reliable in the lower jaw in our study, which was similar to the studies by Toodehzaeim et al, [19] and Marchionni et al [20]. The higher frequency of dental anomalies in the upper jaw could be the reason for this difference.

## CONCLUSION

The prediction equations obtained in this study can be used to predict the mesiodistal width of unerupted lateral incisors, canines and premolars more accurately in the Iranian population in early mixed dentition period without using radiographs, which were more valid in the lower jaw and in males.
